# Multi-layered control of PD-L1 expression in Epstein-Barr virus-associated gastric cancer

**DOI:** 10.20517/2394-4722.2020.12

**Published:** 2020-05-23

**Authors:** Christos N. Miliotis, Frank J. Slack

**Affiliations:** HMS Initiative for RNA Medicine, Department of Pathology, Cancer Center, Beth Israel Deaconess Medical Center, Harvard Medical School, Boston, MA 02115, USA.

**Keywords:** Epstein-Barr virus, gastric cancer, immunotherapy, programmed death-ligand 1

## Abstract

Gastric cancer (GC) is the fifth most common cancer worldwide. In approximately 10% of GC cases, cancer cells show ubiquitous and monoclonal Epstein-Barr virus (EBV) infection. A significant feature of EBV-associated GC (EBVaGC) is high lymphocytic infiltration and high expression of immune checkpoint proteins, including programmed death-ligand 1 (PD-L1). This highlights EBVaGC as a strong candidate for immune checkpoint blockade therapy. Indeed, several recent studies have shown that EBV positivity in GC correlates with positive response to programmed cell death protein 1 (PD-1)/PD-L1 blockade therapy. Understanding the mechanisms that control PD-L1 expression in EBVaGC can indicate new predictive biomarkers for immunotherapy, as well as therapeutic targets for combination therapy. Various mechanisms have been implicated in PD-L1 expression regulation, including structural variations, post-transcriptional control, oncogenic activation of intrinsic signaling pathways, and increased sensitivity to extrinsic signals. This review provides the most recent updates on the multilayered control of PD-L1 expression in EBVaGC.

## INTRODUCTION

Gastric cancer (GC) is the fifth most common and third most deadly cancer worldwide^[1]^. Due to the lack of early symptoms, patients are usually diagnosed with locally advanced or metastatic cancer, when conventional lines of therapy are less effective. The 5-year survival rate for GC in the United States varies by stage and ranges from 68% in patients with localized cancer to 5% in patients with distant metastases^[2]^.

The majority of GCs (90%−95%) are adenocarcinomas^[3]^. The Cancer Genome Atlas (TCGA) recently undertook a comprehensive molecular characterization of hundreds of gastric adenocarcinomas and proposed classification of GC into four molecular subtypes: Epstein-Barr virus (EBV)-positive, microsatellite instability (MSI), chromosomal instability (CIN), and genomically stable (GS) subgroups^[4]^. EBV-positive tumors are characterized by DNA hypermethylation, frequent phosphatidylinositol-4,5-bisphosphate 3-kinase catalytic subunit alpha (*PIK3CA*) mutations, and programmed cell death ligand 1 (PD-L1)/programmed cell death ligand 2 (PD-L2)/Janus kinase 2 (JAK2) overexpression; MSI tumors have high mutation and DNA methylation rates; CIN tumors contain chromosomal alterations affecting mainly tyrosine kinase receptors; and finally, GS tumors are chromosomally stable and have a high frequency of cadherin 1 (*CDH1*) and Ras homolog family member A (*RHOA*) mutations [Table 1]^[4]^. This review will focus on EBV-positive tumors.

EBV is a double-stranded DNA virus that belongs to the herpesvirus family. An estimated 90% of the human population show signs of previous infection with EBV^[7]^. The virus is usually transmitted orally through saliva. Primary infection is most commonly asymptomatic, but it can lead to acute mononucleosis in a subset of the population, primarily adolescents and young adults^[8]^. Following primary infection, the virus establishes a lifelong latent infection in the host. EBV can remain latent in both lymphocytes and epithelial cells, where it expresses only a subset of its genes. Depending on which viral genes are expressed, latent EBV infection is typically classified into four latency programs, known as latency 0, I, II, and III [Table 2]^[7]^.

In 1964, EBV was the first human virus to be associated with cancer, when it was discovered in Burkitt’s Lymphoma^[9,10]^. Since then, EBV has also been linked to other lymphomas, including Hodgkin lymphoma, diffuse large B-cell lymphoma (DLBCL), and Natural Killer/T-cell lymphomas (NK-T lymphomas)^[11]^. In addition, EBV has been associated with certain epithelial cancers, notably Nasopharyngeal Carcinoma (NPC) and GC. In both lymphoid and epithelial cancers, EBV persists in a latent state in infected cells^[11]^. However, different EBV-associated cancers demonstrate different viral gene expression patterns [Table 2].

In EBV-associated GC (EBVaGC), the virus expresses all latency I products, meaning EBV nuclear antigen 1 (EBNA1), Epstein-Barr encoding region small noncoding RNAs, BamHI A rightward transcripts (BARTs), and BART miRNAs, while around 50% of the cases also show Latent Membrane Protein 2A (LMP2A) expression, which is typically associated with latency II^[12]^. LMP1, another latency II protein, is typically not detected in EBVaGC samples^[12–14]^. There is strong geographical variation in the prevalence of EBVaGC but overall around 10% of gastric adenocarcinomas worldwide are classified as EBV-positive^[15]^.

In EBVaGC, the EBV genome is mainly maintained as a nuclear episome and cancer cells within the tumor show ubiquitous and monoclonal EBV infection^[12,16]^. The monoclonality of EBV infection suggests the clonal selection of virus-infected cells in early stages of cancer development. The role of EBV in gastric carcinogenesis is still under investigation, but EBV infection is thought to contribute to GC progression or maintenance, both directly through the activity of viral proteins or RNAs and indirectly through the induction of somatic alterations in the host genome and epigenome^[17]^.

## PD-L1 EXPRESSION IN EBVAGC

Multiple studies have shown that EBVaGC is commonly characterized by high lymphocytic infiltration in the tumor microenvironment, coupled with overexpression of immune-related genes, including *PD-L1* (also known as *CD274*)^[4,5,18]^. PD-L1 is a glycoprotein, expressed by both cancer cells and stromal immune cells in the tumor, that engages the programmed cell death 1 (PD-1) receptor expressed on the surface of infiltrating cytotoxic T cells (CTLs)^[19]^. The interaction between PD-L1 and PD-1 leads to the inhibition of the tumor-infiltrating CTLs, preventing them from attacking and eliminating tumor cells. *PD-L1* is only one of multiple immune checkpoint genes that are known to be upregulated in EBV-positive compared to EBV-negative cancers. *PD-L2*, Lymphocyte activation gene-3 (*LAG3*), T cell immunoglobulin and mucin domain (*Tim-3*), Cluster of differentiation 80 (*CD80*), Cluster of differentiation 86 (*CD86*), and Indoleamine 2, 3-dioxygenase 1 (*IDO1*) are also upregulated, but *PD-L1* has received particular interest because the PD-1/ PD-L1 axis is the target of some recent breakthrough cancer therapies^[20]^. Monoclonal antibodies that block the interaction between PD-L1 and PD-1, thus restoring the ability of the immune system to surveil and attack the tumor, have shown promising results as therapeutic agents against multiple cancers, including non-small cell lung cancer and melanoma^[19]^. Recently, the Food and Drug Administration (FDA) approved pembrolizumab, a mAb targeting PD-1, as a third-line therapy for advanced gastric tumors that are positive for PD-L1 expression based on immunohistochemical (IHC) staining^[21,22]^.

The clinical efficacy and adverse effects of PD-1/PD-L1 therapy vary tremendously among patients. High expression of PD-L1 in the tumor has been implicated as a significant predictive biomarker for positive response to PD-1/PD-L1 therapy^[23]^. However, several clinical studies have demonstrated that some tumors with high PD-L1 expression do not respond to PD-1/PD-L1 therapy, while some tumors with moderate or low PD-L1 expression do show beneficial responses. This discrepancy could be attributed in part to the fact that direct determination of PD-L1 expression in the tumor, which is typically performed by IHC assays, has been proven to be difficult and inconsistent^[24,25]^. Understanding the molecular mechanisms that control PD-L1 expression in cancer may give rise to more accurate biomarkers for positive response to PD-1/PD-L1 therapy. In addition, regulators of PD-L1 expression could serve as targets for potential combination therapies.

In a phase II clinical trial, Kim *et al.*^[6]^ identified EBV-positivity and high mutational load as reliable and independent biomarkers for the clinical efficacy of pembrolizumab in GC patients and recommended considering pembrolizumab as an early therapeutic option for EBVaGC. MSI-high (MSI-H) tumors are characterized by high rates of somatic mutations, resulting in increased presentation of neoantigens and thus stimulation of anti-tumor immunity. As is the case in EBV-positive GCs, MSI-H tumors have high levels of tumor-infiltrating lymphocytes (TILs)^[26]^. The FDA has already approved the use of front-line pembrolizumab monotherapy in advanced MSI-H solid tumors of any origin, including stomach^[27]^.

**Figure 1.**
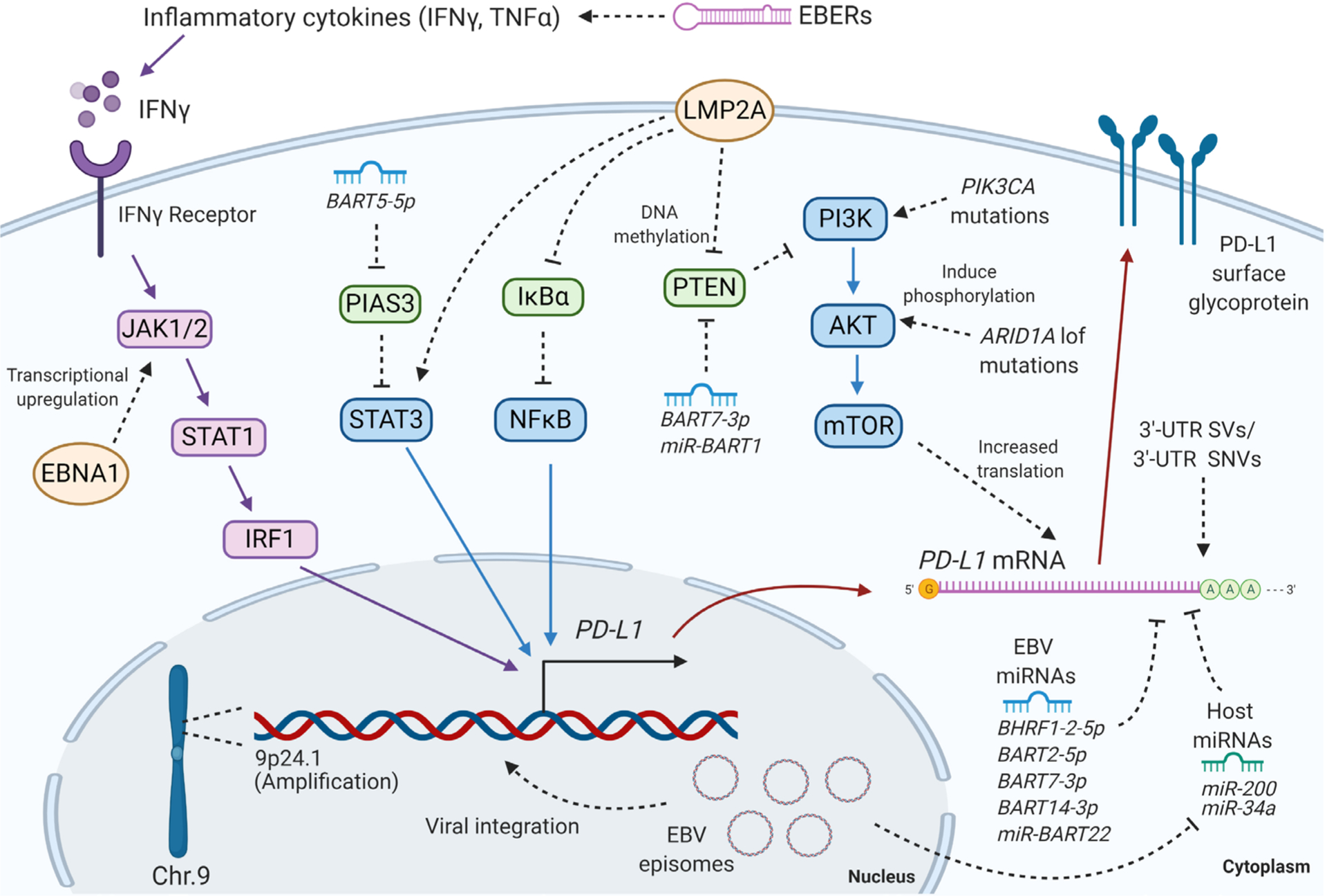
The mechanisms of PD-L1 regulation in EBVaGC discussed in this review. EBV: Epstein-Barr virus; EBVaGC: EBV-associated gastric cancer; PD-L1: programmed cell death ligand 1; IFNγ: interferon gamma; TNFα: tumor necrosis factor alpha; EBER: Epstein-Barr encoding region; JAK: Janus kinase; STAT: signal transducers and activators of transcription; IRF: interferon regulatory factor 1; EBNA1: EBV nuclear antigen 1; BART: BamHI A rightward transcripts; PIAS: protein inhibitor of activated STAT 3; IκBα: nuclear factor of kappa light polypeptide gene enhancer in B-cells inhibitor, alpha; NFκB: nuclear factor kappa-light-chain-enhancer of activated B cells; LMP2A: latent membrane protein 2A; PTEN: phosphatase and tensin homolog; PIK3CA: phosphatidylinositol-4,5-bisphosphate 3-kinase catalytic subunit alpha; PI3K: phosphoinositide 3-kinase; AKT: protein kinase B; ARID1A: AT-rich interaction domain 1A; mTOR: mammalian target of rapamycin; 3′-UTR: 3′ untranslated region; SV: structural variation; SNV: single nucleotide variation; BHRF1: BamHI fragment H rightward open reading frame 1. Created with BioRender.com

EBVaGC appears to employ some common and some unique mechanisms for PD-L1 regulation. This review presents the most recent findings on PD-L1 regulation in EBVaGC and discusses some of the discrepancies in the literature, parallels with other EBV-associated cancers, and questions to be addressed in the future [Figure 1].

## EVOLUTIONARY PRESSURE FOR PD-L1 OVEREXPRESSION

EBVaGC shows high levels of TILs and thus is under strong evolutionary pressure for the development of immune evasion strategies^[4]^. One such strategy is PD-L1 overexpression, which is evident in both cancer and stromal immune cells. It is speculated that the high immune activity in the EBVaGC microenvironment reflects the strong immunogenicity of EBV in the body. Up to 5% of the circulating CD8^+^ T cells in EBV-infected individuals are believed to be reactive to lytic or latent EBV antigens^[28]^. Indeed, several groups have detected expression of a subset of lytic genes in EBVaGC^[29–31]^. According to Borozan *et al.*^[29]^, the lytic expression pattern detected in EBVaGC, which includes subsets of both early and late lytic proteins, does not indicate lytic, or abortive lytic, replication. However, the presentation of lytic viral antigens by infected cancer cells might be driving further immune activation. Camargo *et al.*^[32]^ detected higher levels of circulating antibodies targeting both latent and lytic proteins in patients with EBV-positive compared to EBV-negative GCs, even though virtually all patients in the study were seropositive for antibodies against EBNA and the viral capsid. This further suggests that lytic proteins are expressed in EBVaGC and potentially contribute to the activation of the host immune response.

## SOMATIC STRUCTURAL VARIATIONS

Somatic genomic alterations include short variations, such as single nucleotide substitutions and short insertions/deletions (indels), as well as long variations, also known as structural variations (SVs)^[33]^. SVs affect large chromosomal regions (longer than 50 base-pairs) and include amplifications, deletions, inversions, and translocations^[33]^. Somatic changes occur continuously in the life of an individual and are usually repaired, but their accumulation over time can contribute to carcinogenesis^[33]^. EBVaGC only appears in a small percentage of EBV-infected individuals and typically long after primary EBV infection suggesting that somatic genomic changes are likely required for cancer development^[34]^.

### Gene amplification

Recent high-throughput sequencing studies have revealed different genomic alterations that lead to PD-L1 overexpression in EBVaGC. TCGA performed a somatic copy-number aberrations analysis in gastric adenocarcinoma samples and identified a frequent (15% of EBVaGC cases) somatic focal amplification at the chromosomal region 9p24.1, which includes the genes *PD-L1*, *PD-L2*, and *JAK2*^[4]^. This amplification was significantly more common in EBV-positive than EBV-negative tumors. Importantly, they showed that the 9p24.1 amplification correlated positively with *PD-L1* mRNA levels in the tumor^[4]^. Saito *et al.*^[35]^ detected 9p24.1 amplification events at a similar rate in a different EBVaGC cohort and demonstrated a direct association between *PD-L1* copy number and protein expression. Using fluorescence in-situ hybridization and IHC staining, they showed that *PD-L1* genomic amplifications were specifically detected in cancer cells that showed high PD-L1 protein expression. Similar amplification patterns have been detected in other cancers, including hepatocellular carcinomas (HCC) and DLBCL^[23,36]^.

### Integration of EBV to the host genome

In EBVaGC, the EBV genome is mostly maintained in the nucleus as a circular episome^[16]^. Some studies have also reported viral integration events, even though they are considered rare^[4,37,38]^. TCGA identified the presence of RNA sequencing (RNAseq) reads corresponding to a fusion between the host *PLGRKT* gene and the EBV gene *BHLF1* in one EBVaGC sample (FP-7998–01)^[4]^. This is notable given that the *PLGRKT* lies within the 9p24.1 chromosomal region and is adjacent to the *PD-L1* locus. It was later shown in the same sample, that the amplified region containing the integrated EBV genome (Copy Number = 4) has a breakpoint in the *PD-L1* 3’ untranslated region (3’-UTR)^[37]^. Xu *et al.*^[38]^ performed a genome-wide EBV integration analysis in multiple malignancies and identified that EBV integrates primarily in unstable chromosomal regions of the host genome. They identified EBV integration events in 25% (10/39) of the GC samples analyzed, with some of the integration breakpoints mapping close to known tumor suppressor genes^[38]^. None of their samples had integrated virus close to *PD-L1*, but studies with bigger EBVaGC sample sizes are necessary to identify the frequency and significance of viral integration in or close to the *PD-L1* locus. Viral integration in the host genome has been associated with increased PD-L1 expression in other virus-associated cancers. Cao *et al.*^[20]^ identified integrated HPV genomes in the *PD-L1* or *PD-L2* loci in three cases from the TCGA Head and Neck Squamous Cell Carcinoma cohort and showed that these integration events correlated with elevated PD-L1 and PD-L2 expression.

### 3’-UTR structural variations

SVs in the *PD-L1* 3’-UTR have also been associated with increased PD-L1 expression in EBVaGC^[34,37]^. Kataoka *et al.*^[37]^ analyzed RNAseq data from all TCGA cancer types and searched for 3’-UTR disruptions in *PD-L1*. The authors identified *PD-L1* 3’-UTR truncations in 31/10,210 cancer cases and showed that they correlated with high PD-L1 expression. The highest frequency of 3’-UTR truncations was found in DLBCL (4/48) and GC (9/415), with a third of the GC samples (3/9) being EBV-positive. Therefore, around 10% of EBVaGC samples in TCGA were found to have *PD-L1* 3’-UTR SVs^[34,37]^. In a follow-up study, Kataoka *et al.*^[34]^ analyzed samples from multiple EBV-associated lymphomas and found that *PD-L1* 3’-UTR SVs were significantly more common in EBV-positive compared to EBV-negative lymphomas. They report that *PD-L1* 3’-UTR genomic truncations in cell lines and mouse models promote PD-L1 overexpression and immune evasion, consistent with the patient data^[37]^.

## POST-TRANSCRIPTIONAL REGULATION

The 3’-UTR contains sequences or structural regions, called regulatory elements, that are important for the post-transcriptional regulation of a gene. These regulatory elements control binding to miRNAs and RNA-binding proteins (RBP), which influence mRNA stability, translation rate, and localization^[39]^. miRNAs are short non-coding RNAs that silence gene expression by binding to complementary sequences in the 3’-UTR of target mRNAs. miRNA-mRNA binding usually triggers mRNA degradation or blocks translation. The fact that 3’-UTR shortening has such a profound effect on PD-L1 expression in multiple cancers indicates that PD-L1 is under tight post-transcriptional control^[37]^.

### 3’-UTR short variations

Mutations in the 3’-UTR have the capacity to remove existing or create new binding sites for miRNAs and RBPs. Some germline and somatic mutations in the 3’-UTR of *PD-L1* have been shown to correlate with PD-L1 expression in gastric and other cancers^[40–44]^. Wu *et al.*^[43]^ analyzed 728 GC samples and found that the AA and AG genotypes in rs2297136, a germline single nucleotide polymorphism (SNP) located in the 3’-UTR of *PD-L1*, were associated with lower PD-L1 protein levels. They reported that the miRNAs miR-324–5p and miR-362 are predicted to bind to that region of the *PD-L1* 3’-UTR, but no validation experiments were pursued. Wang *et al.*^[44]^ polymerase chain reaction (PCR)-amplified and sequenced the 3’-UTR of *PD-L1* in hundreds of GC and matched normal samples and identified a frequent guanineto-cytosine somatic mutation that correlated with increased PD-L1 protein expression. It was shown that this mutation maps to a seed-binding region for miR-570 and it was proven experimentally that it increases PD-L1 expression by disrupting miR-570 binding^[44]^. To date, most studies looking at *PD-L1* 3’-UTR mutations have been low-throughput, with small sample sizes or targeted on specific SNP locations. There has not been a comprehensive study looking at the frequency and effect of all possible somatic and germline variants in the *PD-L1* 3’-UTR in EBVaGC or other EBV-associated cancers. The fact that SVs in the *PD-L1* 3’-UTR appear to occur more frequently in EBV-positive than EBV-negative cancers raises the question of whether short variants in the 3’-UTR could be an alternative or parallel mechanism for PD-L1 overexpression. Large-scale variant calling studies in gastric and other cancers, including the TCGA somatic mutation data, have mostly relied on whole exome sequencing data and exclude 3’-UTR sequences. This has created a gap in our understanding of 3’-UTR variations in cancer in general.

### Host miRNAs and RBPs

Multiple miRNAs have been implicated in the control of PD-L1 expression in GC^[45]^. Some miRNAs, such as miR-152 and miR-200, target the 3’-UTR of PD-L1 directly. Other miRNAs, such as miR-19a and miR-19b, affect PD-L1 levels indirectly, by targeting signaling pathways or transcription factors that regulate PD-L1 expression^[45–48]^.

EBVaGC has been reported to exhibit a distinct host miRNA expression profile from other GC subtypes^[4,49,50]^. Differentially expressed miRNAs include ones that have been independently shown to target PD-L1, with the most notable example being miR-200^[48]^. miR-200 is a family of miRNAs found in two distinct genomic clusters and consists of miR-200a, miR-200b, miR-429, miR-200c, and miR-141^[51]^. miR-200a, miR-200b, and miR-429 form a cluster on chromosome 1, while miR-200c and miR-141 form a cluster on chromosome 12. miR-200b, miR-200c and miR-429 share the same seed sequence, while miR-200a and miR-141 have a seed sequence that differs from the others by one nucleotide. The *PD-L1* 3’-UTR contains one binding site for each seed-sharing functional cluster of miR-200 miRNAs and all miR-200a, b, and c have been shown to directly silence PD-L1 expression^[48]^.

The miR-200 family has been shown to be downregulated in EBV-positive compared to EBV-negative GC samples, as well as in EBV-negative GC cells following infection with recombinant EBV (rEBV) *in vitro*^[52,53]^. Whether or how EBV latent proteins downregulate miR-200 remains unclear. Shinozaki *et al.*^[53]^ reported that overexpression of any of *EBNA1*, *LMP2A*, or *BARF0* (a *BART* transcript) in EBV-negative GC cells (MKN74 cell line) leads to miR-200a/b transcriptional repression, while overexpression of EBERs downregulates the mature miRNAs post-transcriptionally. The authors concluded that EBV latent proteins and RNAs act synergistically to downregulate miR-200. This is in contrast with a study by Marquitz *et al.*^[52]^ on a different EBV-negative GC cell line, AGS. They reported that rEBV-infected AGS (AGS-EBV) cells showed consistent downregulation in some tumor suppressor miRNAs, including miR-200, when compared to the parental uninfected cells. However, *EBNA1* or *LMP1* overexpression in AGS did not affect the expression levels of miR-200 and most of the other EBV-downregulated miRNAs^[52]^. They speculate that EBV-mediated cellular miRNA downregulation might not be mediated by the latent viral proteins, but by viral miRNAs, through their effect on host transcription factors, or by EBERs^[52]^. Another possible mechanism of host miRNA downregulation in EBV-infected cells is methylation of their promoter sequences. EBV infection in GC cells has been shown to promote extensive DNA methylation, partly through the activity of the latent protein LMP2A, which induces DNA methyltransferases (DNMTs)^[54,55]^. Indeed, the miR-200c promoter contains CpG islands that have been shown to be methylated by DNMT3a in GC^[56]^.

Research from our group described an EBV gene/host miRNA/PD-L1 regulation axis in B cell lymphomas, where the EBV protein EBNA2 induces PD-L1 expression by transcriptionally repressing miR-34a, a miRNA that downregulates PD-L1^[57]^. EBNA2 is a latency III-associated protein and thus is not expressed in GC. However, miR-34a has been shown to be downregulated in GC overall and to be transcriptionally repressed by EBNA1 in EBVaGC^[58,59]^.

The *PD-L1* 3’-UTR contains multiple adenosine-uridine (AU)-rich elements (AREs) that are known to serve as binding sites for different RBPs^[60]^. In GC, the RBP tristetraprolin (TTP) has been shown to bind to AREs in the *PD-L1* 3’-UTR and to promote mRNA destabilization, leading to reduced PD-L1 expression^[62]^. In addition, in NPC, the EBV latent protein LMP1 reduces TTP expression through extracellular-signal-regulated kinase 1/2 (ERK1/2) activation^[63]^. Nonetheless, there has not been an association between EBV infection and TTP expression in GC.

### Viral miRNAs

EBV expresses 25 precursor miRNAs (pre-miRNAs) and 44 mature miRNAs. Three pre-miRNAs are derived from the BamHI fragment H rightward open reading frame 1 (BHRF1) region of the viral genome, while the rest are derived from the BART region^[64]^. BART miRNAs are thought to play an important role in EBVaGC and are highly abundant, accounting for up to 15–20% of the total miRNA pool in some EBVaGC cell lines^[65]^. Some studies have demonstrated that xenografts of EBV-infected GC cell lines, including AGS-EBV and SNU719 cells, in immunocompromised mice show up to 10- or 100-fold overexpression of BART miRNAs compared to their parental cell lines^[66,67]^. This suggests that BART miRNAs might be particularly important for cancer progression *in vivo*. BART miRNAs are known to play a range of roles in GC, targeting host cell apoptosis, cell cycle, and metastasis^[68,69]^. Their role in immune evasion is less well studied in GC, although multiple immune-related functions have been attributed to BART miRNAs in other EBV-associated cancers^[64]^.

In contrast to BART miRNAs, which are expressed in all EBV latent stages, BHRF1 miRNAs are thought to be expressed only in latency III and lytic replication. BHRF1 miRNAs are commonly reported as barely detectable in GC tissues and cell lines^[4,54,62]^. However, Treece *et al.*^[49]^ analyzed miRNA expression in FFPE tissues from 78 cases of gastric adenocarcinoma, including 20 EBVaGCs, and reported that BHRF1-2-5p was significantly overexpressed in EBV-infected *vs*. EBV-negative cancers, although to a lower extent than BART miRNAs. Marquitz *et al.*^[52]^ also detected low expression of BHRF1 miRNAs in sequencing from EBV-infected GC cells (AGS-EBV). Both groups attributed the detection of BHRF1 miRNAs to possible low levels of viral replication. Given that several studies have reported the expression of a subset of lytic transcripts in EBVaGC, even in the absence of lytic replication, the level and importance of BHRF1 miRNA expression remain to be determined. In an *in vitro* model of EBV-driven B-cell differentiation, BHRF1-2-5p was found to downregulate PD-L1^[70]^. The same study identified potential binding sites for some BART miRNAs in the *PD-L1* 3’-UTR, including BART19–3p, but overexpression of the miRNA did not appear to influence PD-L1 expression. Four BART miRNAs, BART2–5p, BART7–3p, BART14–3p, miR-BART22 were found to interact with *PD-L1* mRNA in a high-throughput photoactivatable ribonucleoside-enhanced crosslinking and immunoprecipitation (PAR-CLIP) study in lymphoblastoid cell lines, but no validation experiments have been performed^[71,72]^. A recent study reported that miR-BART5–5p, which shares seed homology with the host miRNAs miR-18a-5p and miR-18b-5p, leads to signal transducers and activators of transcription 3 (STAT3)-dependent transcriptional PD-L1 upregulation by targeting the STAT3 inhibitor Protein Inhibitor Of Activated STAT 3 (PIAS3)^[73]^.

## INTRINSIC SIGNALING

PD-L1 expression can be dysregulated by oncogenic activation of signaling pathways like the JAK/STAT, phosphoinositide 3-kinase (PI3K)/protein kinase B (Akt)/mammalian target of rapamycin (mTOR), Mitogen-activated protein kinase/ERK kinase (MEK)/ERK, and Jun/Activator protein 1 (AP-1) pathways. These pathways act independently or synergistically to control PD-L1 expression, at the transcriptional, post-transcriptional, and post-translational stage^[74–76]^. Their importance in PD-L1 regulation tends to vary among different cancer types. The constitutive activation of an intrinsic signaling pathway in cancer is usually the result of mutations or SVs in genes of key components or regulators of the pathway. In the case of virus-associated cancers, viral proteins can also induce constitutive signaling in infected cells.

### Signaling activation by host gene mutations

A frequently mutated gene in EBVaGC cancer is *PIK3CA*, which encodes a catalytic component of the PI3K kinase^[4,77]^. The most common variants in *PIK3CA* are associated with increased PI3K signaling activity^[77]^. The PI3K pathway is thought to regulate PD-L1 expression in a tissue-specific manner. Loss of Phosphatase and tensin homolog (PTEN) leads to activation of the PI3K pathway and induction of PD-L1 expression in gliomas and colorectal cancer^[78,79]^. In gliomas, Parsa *et al.*^[79]^ showed that the PI3K/Akt/ mTOR pathway increases *PD-L1* mRNA translation through polysomal recruitment. In the case of GC, there is conflicting evidence for the importance of the PI3K pathway in PD-L1 regulation. Supporting the importance of PI3K signaling in promoting PD-L1 expression, Kim *et al.*^[80]^ showed that the PI3K inhibitor LY294002 reduced PD-L1 expression in three GC cell lines. In addition, Menyhárt *et al.*^[81]^ performed hierarchical clustering to determine the mutations that could best stratify TCGA GC patients based on PD-L1 expression. They showed that the mutation status of *PIK3CA* served as the best root node for the stratification, while mutations in other immune-related genes like *MEF2C*, *SLC11A1*, and *KIF15* could help further refine it, suggesting potential interactions between these genes for the control of PD-L1 expression^[81]^. However, Mimura *et al.*^[82]^ performed experiments with a panel of GC cell lines and determined that the PI3K inhibitor wortmannin did not affect PD-L1 expression. Besides, Seo *et al.*^[77]^ analyzed 112 EBV-positive GC samples and reported that *PIK3CA* mutations did not show any correlation with PD-L1 expression or TIL abundance. Therefore, the importance of *PIK3CA* mutations and PI3K signaling in the control of PD-L1 expression in GC and EBVaGC is still unclear, and may be dependent on interactions with other mutations and signaling pathways.

Another gene that is commonly mutated in EBVaGC is AT-Rich Interaction Domain 1A (*ARID1A*), which encodes an important component of the SWItch/Sucrose Non-Fermentable (SWI/SNF) chromatin remodeling complex^[4]^. Mutations in *ARID1A* are usually loss-of-function mutations. Kim *et al.*^[80]^ analyzed 273 GC samples and showed that ARID1A protein loss correlated with PD-L1 positivity (as defined by IHC staining), independently of EBV or MSI status. They also showed that *ARID1A* knockdown *in vitro* directly leads to PD-L1 overexpression, through the activation of the PI3K/Akt pathway. To account for the variability in PD-L1 expression among *ARID1A*-mutated tumors, the authors looked for additional mutations that could be acting synergistically with *ARID1A* mutations. They found *KRAS* mutations in the three *ARID1A*-mutated MSI-H tumors with the highest PD-L1 expression, while two other samples that harbored *KRAS* but not *ARID1A* mutations did not show elevated PD-L1 levels. These data further suggest that PD-L1 expression is controlled by multiple oncogenic signaling pathways acting in coordination.

### Signaling activation by viral latent proteins

The EBV latent membrane proteins (LMPs) are potent oncoproteins and are known to activate oncogenic signaling cascades in EBV-associated malignancies. In epithelial cancers, LMP2A has been found to regulate the nuclear factor kappa-light-chain-enhancer of activated B cells (NFκB), PI3K/Akt, MEK/ERK, and transforming growth factor beta (TGFβ) pathways^[83–86]^. In GC specifically, LMP2A has been reported to lead to constitutive NFκB activation by inhibiting the expression of the nuclear factor of kappa light polypeptide gene enhancer in B-cells inhibitor, alpha (IκBα), which negatively regulates NFκB activity^[83,84]^. The mechanism of IκBα downregulation appears to be unrelated to promoter methylation and remains to be determined. Independent studies have shown that NFκB promotes PD-L1 transcription in GC^[87–89]^, but no direct association has been made between LMP2A, NFκB, and PD-L1. As mentioned above, LMP2A is also thought to be important for promoting the DNA hypermethylated state observed in EBVaGC. Hino *et al.*^[90]^ showed that LMP2A induces phosphorylation and activation of STAT3, which in turn leads to overexpression of DNMT1, thus changing the DNA methylation landscape of the cell. They showed that one of the targets of DNMT1 is *PTEN*, which encodes a negative regulator of the PI3K pathway. The hypermethylation of the *PTEN* promoter reduces PTEN expression and leads to overactivation of the PI3K pathway^[90]^. Interestingly, PTEN is also known to be directly targeted by miR-BART1 and BART7–3p in NPC^[91,92]^. Moon *et al.*^[93]^ reported that the small interfering RNA (siRNA)-mediated knockdown of LMP2A did not affect PD-L1 expression in SNU719, an EBVaGC cell line. However, more studies with additional methods of manipulating LMP2A expression and activity are required to elucidate its role in PD-L1 regulation.

## INTERFERON GAMMA-INDUCIBLE PD-L1 EXPRESSION

Multiple studies have reported that GC patients with higher levels of CD8^+^ TILs also have higher PD-L1 expression^[94–96]^. This suggests that PD-L1 overexpression in EBVaGC is at least partly a result of the evolutionary pressure from the adaptive immune response acting against the cancer. When CD8^+^ T cells are activated, such as through the recognition of neoantigens or viral antigens presented by cancer cells, they produce inflammatory cytokines like tumor necrosis factor alpha (TNFα) and interferon gamma (IFNγ)^[97,98]^. IFNγ has been shown to induce PD-L1 expression in various cancers^[99]^. In GC, PD-L1 induction by IFNγ appears to occur mainly through the JAK/STAT/Interferon regulatory factor 1 (IRF1) signaling axis^[82,100–102]^. The extent to which different cancer cells are responsive to IFNγ and the downstream effects of IFNγ exposure vary among cancer types and molecular subgroups within a cancer type.

In TCGA and other GC cohorts, when EBVaGC samples are compared to EBV-negative ones, they demonstrate elevated IFNγ signatures, indicated by higher expression of IFNγ, JAK/STAT signaling components, and several IFNγ-induced genes^[4,103]^. In addition, *in vitro* studies have shown that EBVaGC cell lines induce PD-L1 expression in response to IFNγ to a much higher extent than EBV-negative GC cell lines^[93,104]^. PD-L1 induction in response to IFNγ has also been shown to be significantly elevated in other EBV-associated epithelial malignancies, such as NPC^[105]^. In NPC, the viral protein LMP1 acts synergistically with IFNγ to induce PD-L1 expression through the activation of the JAK3/STAT3, NFκB, and AP-1 signaling pathways. In GC, EBNA1 has been shown to promote IFNγ-induced PD-L1 overexpression. Moon *et al.*^[93]^ showed that *EBNA1* knockdown in SNU719, an EBVaGC cell line, resulted in the transcriptional downregulation of JAK2. *EBNA1* knockdown also resulted in a small but significant reduction in constitutive and IFNγ-induced PD-L1 levels. However, ectopic EBNA1 expression in the EBV-negative GC cell line AGS did not affect constitutive or IFNγ-induced PD-L1 expression. In contrast, Su *et al.*^[106]^ reported that AGS-EBV cells are more sensitive to IFNγ/TNFα treatment, showing higher downstream PD-L1 upregulation, than the uninfected parental cell line. This suggests that other viral factors, in addition to EBNA1, might be necessary for increasing IFNγ-induced PD-L1 expression in EBV-infected GC cells. Further studies are required to determine how EBNA1 and other viral or host factors promote increased IFNγ sensitivity and PD-L1 expression in EBVaGC.

Nakayama *et al.*^[107]^ analyzed 43 EBVaGC samples and showed that the number of EBV genomes per cancer cell (EBV copy number) correlates positively with PD-L1 expression. Similarly, Strong *et al.*^[31]^ performed cellular gene expression analysis on 12 EBV-positive and 20 EBV-negative TCGA GC samples and showed that, following hierarchical clustering, the 4 EBV-positive samples that had a much higher EBV coverage depth (in RNAseq data) than the rest of the EBV-positive samples formed a well-defined gene expression cluster. When they compared expression between “high” and “low” EBV GC samples, a large proportion of the genes that were upregulated in the “high” EBVaGC group were immune-related, including IFNγ, STAT1, IRF1, and multiple IFNγ-induced genes^[31]^. As the authors state, EBERs, which have been shown to induce IFNγ and TNFα production in peripheral blood mononuclear cells *in vitro*, could be contributing to the elevated IFNγ signature found in “high” compared to “low” EBV GC samples^[108]^.

## CONCLUSION

Even though GC is declining in the United States, it still has one of the lowest 5-year survival rates of any cancer type^[2]^. This highlights the need for new therapeutic strategies, especially for metastatic cases that have the poorest prognosis and account for most of the new diagnoses every year. The molecular heterogeneity of GC correlates with the response rate to different therapies, indicating that different approaches should be considered for different molecular subgroups. The FDA recently approved pembrolizumab as second-line therapy for patients with advanced MSI-H tumors of any type, including GC^[109]^. In the last few years, several molecular and clinical studies present EBVaGC as another subgroup of GC that could benefit from early-line treatment with immune checkpoint inhibitors^[6,107]^.

In EBVaGC, high immune activation in the tumor microenvironment likely acts as a driving force for the selection of immune escape mechanisms such as PD-L1 overexpression. Different mechanisms act independently or synergistically to induce PD-L1 expression. These include somatic genomic modifications, oncogenic activation of intrinsic signaling pathways, increased sensitivity to PD-L1 inducing signals from the tumor microenvironment, and post-transcriptional control mechanisms. Overall, the regulation of PD-L1 expression in EBVaGC is poorly understood and further studies are necessary to explain how EBV and host factors contribute to it. Insight into the important genetic and epigenetic factors that control PD-L1 expression in EBVaGC and other cancers could reveal new biomarkers for positive response to immunotherapy, as well as novel therapeutic targets.

## Figures and Tables

**Table 1.** Immunological and molecular characteristics of GC molecular subtypes

Molecular subtype	EBV-positive	MSI	CIN	GS
Frequency (TCGA STAD) %^[4]^	9	22	50	20
TIL abundance^[5,6]^*	High	High	Low	Low
PD-L1 expression^[4–6]^*	High	High	Low	Low
Somatic mutations^[4]^	Standard rate of mutations (non-hypermutated) Common *PI3KCA*, *ARID1A* mutations	Hypermutated Recurrent mutations in *TP53*, *KRAS*, *ARID1A*, *PIK3CA*, *ERBB3*, *PTEN* and *HLA-B*	Non-hypermutated Common *TP53* mutations	Non-hypermutated Common *RHOA*, *CDH1* mutations
Other molecular Characteristics^[4]^	Hypermethylation (EBV-CIMP, frequent *CDKN2A* silencing) Amplification in 9p24.1 chromosomal region *(PD-L1/PD-L2/JAK2)*	Hypermethylation (MSI-associated gastric CIMP, frequent *MLH1* silencing)	Gene amplifications,frequent in TKRs *EEGFR, VEGFA)* and deletions	

* “High” indicates that the corresponding molecular subtype is typically associated with high levels of TIL or PD-L1, respectively. “Low” indicates that the corresponding molecular subtype is not typically associated with high levels of TIL or PD-L1, respectively. MSI: microsatellite instability; CIN: chromosomal instability; GS: genomically stable; TCGA: the cancer genome atlas; STAD: stomach adenocarcinoma; TILs: tumor infiltrating lymphocytes; PD-L1: programmed death-ligand 1; PIK3CA: phosphatidylinositol-4,5-bisphosphate 3-kinase catalytic subunit alpha; ARID1A: AT-rich interaction domain 1A; TP53: tumor protein p53; KRAS: Kirsten rat sarcoma viral oncogene homolog; ERBB3: Erb-B2 receptor tyrosine kinase 3; PTEN: phosphatase and tensin homolog; HLA-B: human leukocyte antigen B; RHOA: ras homolog family member A; CDH1: cadherin 1; CIMP: CpG island methylator phenotype; CDKN2A: cyclin dependent kinase inhibitor 2A; JAK2: Janus kinase 2; MLH1: MutL homolog 1; TKR: tyrosine kinase receptor; EGFR: epidermal growth factor receptor; VEGFA: vascular endothelial growth factor A; GC: gastric cancer

**Table 2.** Viral genes expressed in each latency program

Latency program	EBV gene expression pattern	Cancer*
0	No viral proteins expressed	
I	EBER 1 & 2, BART miRNAs, EBNA1	BL
	*Latency I* +/− LMP2A	GC
II	*Latency I* + LMP1, LMP2A, LMP2B	NPC, NK-T lymphomas, HL
III	*LatencyII* + EBNA2, EBNA3s, EBNA-LP, BHRF1 miRNAs	PTDL, AIDS-related lymphomas (IB-DLBCL)

* This column indicates some cancers that are typically associated with each latency program. Within each cancer type (e.g., BL and DLBCL) there might be subtypes that show different patterns of latent expression. EBER: Epstein-Barr encoding region; BART: BamHI A rightward transcripts; miRNA: microRNA; BHRF1: BamHI fragment H rightward open reading frame 1; EBNA1: EBV nuclear antigen; LMP: latent membrane protein; EBNA-LP: EBNA leader protein; BL: Burkitt’s lymphoma; GC: gastric cancer; NPC: nasopharyngeal carcinoma; DLBCL: diffuse large B-cell lymphoma; NK-T lymphomas: natural killer/T-cell lymphomas; HL: Hodgkin lymphoma; PTDL: post-transplant lymphoproliferative disorder; IB-DLBCL: immunoblastic DLBCL; EBV: Epstein-Barr virus
